# Time-Series modeling for predicting mortality risk in intensive care unit patients with pulmonary inflammation

**DOI:** 10.3389/fmed.2026.1852032

**Published:** 2026-09-03

**Authors:** Yihai Zhai, Ruixin Xu, Haisu Lu, Siying Lv, Liqin Mo

**Affiliations:** The First Affiliated Hospital of Guangxi Medical University, Nanning, China

**Keywords:** infection, intensive care unit, machine learning, mortality, pneumonia

## Abstract

**Objective:**

This study aims to evaluate dynamic changes in pneumonia-related mortality risk among ICU patients using time-series models and develop interpretable predictive models for early mortality assessment.

**Methods:**

Data were obtained from the MIMIC-IV and eICU Collaborative Research Database. The MIMIC-IV cohort was split into training and testing sets (7:3 ratio), with external validation conducted using the eICU cohort. Missing data were handled using forward filling and multiple imputation. LSTM was used to extract temporal features, which were then entered into five machine-learning classifiers: logistic regression, random forest (RF), Light Gradient Boosting Machine, extreme gradient boosting, and multilayer perceptron. Hyperparameter tuning was performed using cross-validation and grid search. Model performance was assessed using accuracy, precision, recall, F1 score, balanced accuracy, Matthews correlation coefficient (MCC), AUC, Brier score, and Youden's index. The best-performing model was interpreted using Shapley Additive Explanations and Local Interpretable Model-agnostic Explanations.

**Results:**

A total of 4,205 patients were included, including 2,751 patients from MIMIC-IV and 1,454 patients from the eICU cohort. The 7-day mortality rates were 7.92% and 11.69%, respectively. The hybrid LSTM-RF model performed exceptionally, achieving an accuracy of 0.961, precision of 0.938, recall of 0.823, F1 score of 0.874, AUC of 0.940, Balanced ACC of 0.914, and MCC of 0.862. The LSTM-RF model yielded a Brier score of 0.020 and Youden's index of 0.818. Validation results showed an AUC of 0.826, a Brier score of 0.070, and a Youden's index of 0.590.

**Conclusion:**

Integrating random forest with LSTM-derived features improves early mortality risk prediction in ICU patients with pneumonia.

## 1 Introduction

The Intensive Care Unit (ICU) provides specialized medical care for individuals facing life-threatening health conditions. In spite of considerable progress in the domain of critical care, the incidence of infections in the ICU remains 5–10 times higher than that in general hospital wards, with secondary pneumonia presenting an especially severe hazard (1, 2). Frequently arising from progression of underlying diseases or contact with hospital-derived pathogenic microorganisms, secondary lung infection counts as one of the most prevalent and deadly adverse events among ICU patients and correlates with prolonged hospital stays and raised risk of death (3, 4). Given the essential role of the lungs in the process of respiratory gas exchange, pulmonary function disruption disturbs the balance of oxygen and carbon dioxide, bringing about the buildup of metabolic byproducts and physiological strain that could develop into acute respiratory decompensation or systemic sepsis (5, 6). Roughly 25%–30% of ICU patients suffering from pulmonary infection experience mortality within seven days (7), highlighting the requirement for precise predictive evaluation of patient outcomes.

Prior scholarly inquiries have uncovered a range of predictive factors, encompassing age, comorbidities, immune responses, and disease severity (8–10). The complicated interdependencies of these parameters pose substantial barriers to precise prognosis prediction. Widely employed assessment instruments, such as CURB-65, CRB-65, the Pneumonia Severity Index (PSI), and the Quick Sequential Organ Failure Assessment (qSOFA), have notable shortcomings when applied to cohorts within intensive care units (11–13). Individuals suffering from critical illnesses frequently present with multifaceted clinical states that can shift abruptly and without prior warning. Certain patients exhibit only modest physiological deviations at the point of ICU admission, yet subsequently experience deterioration of oxygenation, hemodynamic unsteadiness, exaggerated inflammatory responses, coagulation dysfunctions, metabolic disruptions, or organ impairment. As these alterations advance over the course of ICU care, scoring frameworks rooted in a single time point may fail to sufficiently reflect the advancement of pathological conditions (14). Dependence on initial evaluation exclusively may thus miss early indicators of later clinical worsening, postpone therapeutic intervention, and exert negative impacts on clinical results. Prompt recognition of individuals with elevated mortality risk continues to represent a foremost difficulty within the domain of critical care management.

Rapid advances in artificial intelligence have furnished promising methodologies for the addressing of these aforementioned difficulties (15, 16). Computational learning frameworks have undergone growing deployment for the prediction of mortality among intensive care unit patients with conditions such as acute heart failure (17), acute respiratory distress syndrome (18), and stroke (19). While investigations tailored to specific pathologies and patient populations, comprising those centered on COVID-19 pneumonia, have made substantial progress (20, 21), a comparatively limited number of works have investigated dynamic mortality forecasting among non-COVID intensive care unit patients affected by pneumonia, especially pneumonia associated with underlying diseases or pathogens acquired within hospital settings. The majority of currently available prediction frameworks depend on metrics collected at an individual time point, and as a consequence are unable to fully obtain representations of changing clinical progression pathways. In the same vein, even though certain research efforts have placed attention on age-defined patient groups, their largely static analytical structures impose restrictions on the timely and accurate prediction of worsening clinical status (22–24).

In addition, intensive care unit data repositories are frequently characterized by severe class disproportion, with the count of non-surviving patients being drastically diminished in comparison with that of surviving individuals. This skewed distribution may lead traditional machine learning frameworks to exhibit bias toward the dominant category, decreasing detection sensitivity for fatality instances and undermining the dependability of the constructed model. Clinical integration further necessitates that medical practitioners grasp the rationale underlying model predictions, highlighting the significance of model explainability (25). The construction of a prediction framework that takes into consideration the dynamic advancement of pathological conditions while preserving elevated predictive precision and explanatory capacity consequently remains a major challenge in the domain of intensive care unit prognostic investigation.

For the purpose of tackling the aforementioned constraints, the present investigation sets out to construct a machine learning framework for forecasting of mortality risk among intensive care unit patients suffering from pneumonia, placing emphasis on class imbalance, the temporal advancement of pathological conditions, and the property of interpretability. In contrast to dependence on a sole static evaluation, the put forward system identifies high-risk clinical features observed during the seven days preceding death, enabling earlier identification of worsening pathways. Through combination of electronic health record datasets obtained at multiple timepoints with explainable machine learning approaches, the algorithm is designed to facilitate prompt and evidence-based clinical decision formulation in acute care scenarios and eventually enhance the clinical results of patients.

## 2 Methods

### 2.1 Study population and outcomes

The training assemblage for the prediction of death risk among intensive care unit patients suffering from pneumonia has been procured from MIMIC-IV version 2.2, which encompasses clinical documentation of 73,181 patients receiving inpatient care at Beth Israel Deaconess Medical Center across the period from 2008 to 2019. Access authorization is provided to persons who finish the mandatory PhysioNet (26). This assemblage has been labeled as Data A. The external verification dataset has been sourced from the eICU Collaborative Research Database, which comprises over 200,000 intensive care unit admissions throughout the United States between 2014 and 2015. It furnishes thorough particulars of vital signs, care protocols, illness severity metrics, diagnostic records, and therapeutic interventions, and has been designated as Data B (27). Individuals with pneumonia have been recognized via the diagnostic coding framework unique to each data repository. Within MIMIC-IV, pneumonia cases have been determined through ICD-10 codes J12–J15 and J18, covering viral lower respiratory tract infection, bacterial lung infection, and pneumonia induced by an unclassified pathogen. Codes J16 and J17 have been ruled out, as J16 signifies pneumonia triggered by other infectious agents and may incorporate more varied or non-typical causative origins, while J17 represents pneumonia occurring in disorders categorized under other headings and may mirror secondary lung infection linked to pre-existing health conditions. As the eICU database employs ICD-9 codes, matching ICD-9 pneumonia coding entries have been utilized to uphold alignment with the MIMIC-IV diagnostic standard. Neither data repository incorporated patients with COVID-19-associated pneumonia, as both were gathered prior to the onset of the COVID-19 global outbreak.

Inclusion criteria: (1) ICU stay duration ≥ 24 h; (2) age ≥18 years; (3) pneumonia diagnosis identified using database-specific diagnosis codes; (4) available longitudinal clinical records after completeness filtering. Exclusion criteria: (1) missing key vital-sign variables, such as body temperature, blood pressure, breathing, heart rate; (2) repeated ICU admissions.

### 2.2 Dataset Generation

**Figure 1:**
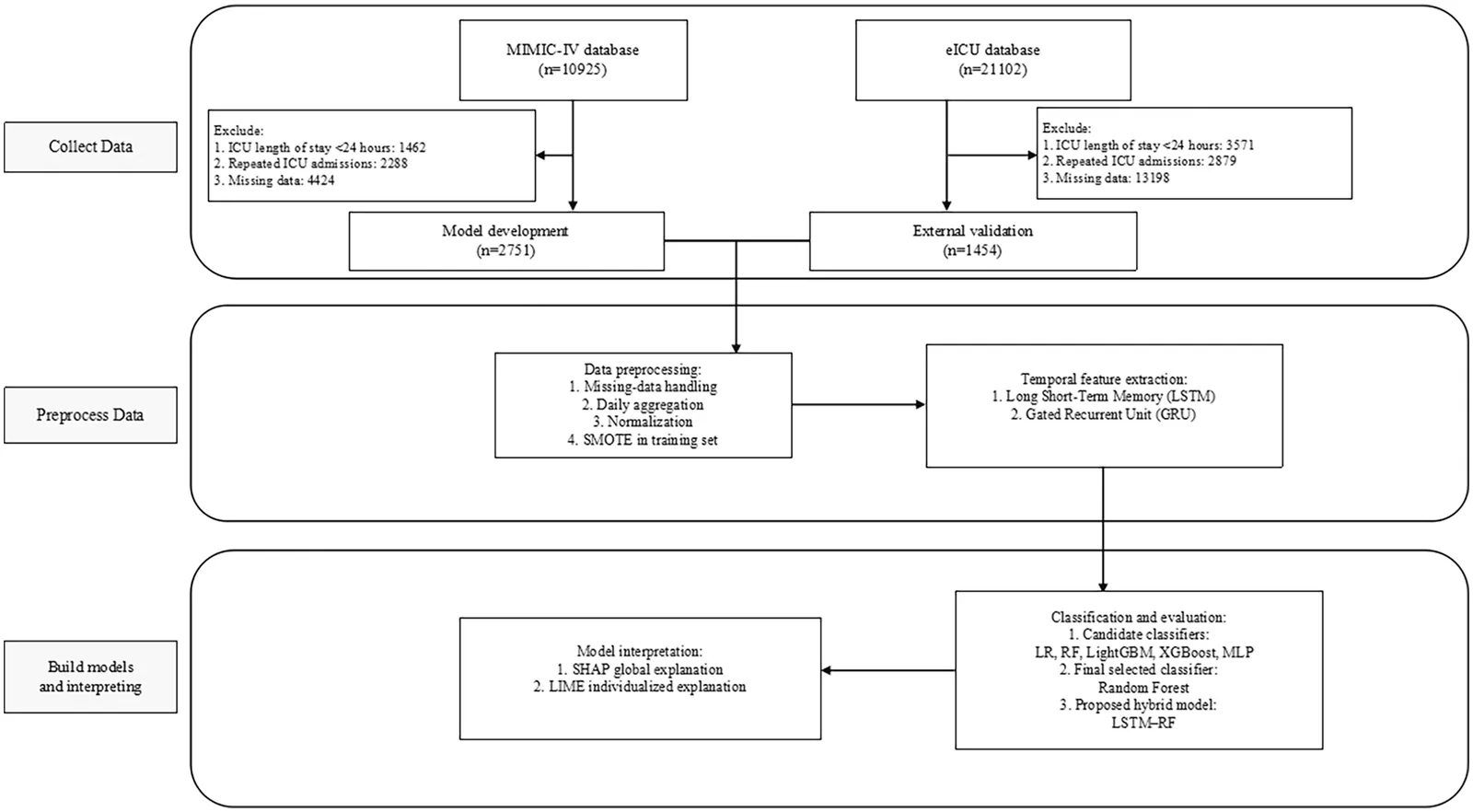
Patient selection flowchart and study design routes.

1. Patients diagnosed with pneumonia were identified using relevant diagnosis codes, initially including 10,925 patients in Data A and 21,102 in Data B.
2. Death or discharge dates were used as reference points for temporal alignment. For each patient, clinical data were extracted from the seven days preceding the reference date and organized into three dynamic windows: 5–7 days, 3–5 days, and ≤3 days before the outcome event. These windows were labeled as dead 1, dead 2, and dead 3, respectively, to characterize the temporal evolution of clinical indicators and to assess short-term all-cause mortality risk among ICU patients with pneumonia.
3. Features were categorized as static or dynamic based on temporal variability. Static features remained constant, while dynamic features were aggregated based on specific time windows. the dynamic variables of the surviving patients were calculated as follows: Dynamic clinical variables were summarized as daily mean values within a retrospective 7-day window before the outcome event. In this study, day 1 referred to the earliest day within the retrospective window, whereas day 7 referred to the day closest to the outcome event. By using daily averages, the model was able to capture longitudinal trends in clinical status while reducing the impact of short-term variability and improving data consistency across time points. For patients with early confirmed mortality, the mean value of the relevant variables is calculated from the 24-hour period preceding the time of death; all subsequent observations are coded as missing (NA). After filtering for data completeness, 2,751 patients in Data A and 1,454 patients in Data B remained, each with clinical outcomes (mortality status) and event times recorded. Figure 1 illustrates the patient enrollment and study design process.

### 2.3 Variable selection and preprocessing

Variables were selected based on clinical relevance and dataset characteristics, with careful consideration of missing data. Static variables included gender, age, history of hypertension, chronic obstructive pulmonary disease, diabetes, and lung cancer. Dynamic variables encompassed vital signs such as diastolic and systolic blood pressure, Diastolic Blood Pressure, temperature, respiration rate, and heart rate. Laboratory results included oxygen saturation, white blood cells, red blood cells, albumin, creatinine, electrolytes (potassium, sodium, chloride, calcium), platelets, liver enzymes (ALT, AST), hemoglobin, activated partial thromboplastin time (APTT), serum bilirubin, international normalized ratio (INR), glucose, lactate dehydrogenase (LDH), and respiratory support type, totaling 30 variables.

Missing longitudinal data were processed at the patient level. For each dynamic variable, patients with no recorded value for that variable across all available time points were excluded, whereas the variable was retained in the dataset. For patients with partially observed longitudinal measurements, within-patient forward filling was first applied to impute missing values using the most recent previous measurement. Missing values that remained after forward filling were subsequently imputed using multiple imputation by chained equations with the mice package in R. After completion of the imputation procedure, continuous variables were normalized prior to model training. Categorical variables, such as gender, hypertension history, and respiratory support type, were encoded as binary values (1 for positive, 0 for negative). Continuous variables, including laboratory results and vital signs, were averaged over the observation period. The study's results were reported in accordance with guidelines for developing and publishing machine learning predictive models in biomedical research (28–30).

### 2.4 Handling class imbalance

Following the elimination of subjects experiencing premature death occurrences, the 7-day fatality proportion within the Data A cohort reached 7.92%, matching a positive-vs-negative specimen proportion of roughly 1:13. This category disproportion could introduce skewing to computational frameworks tuned for general prediction precision toward the dominant group, restricting their capability to capture regularities linked to the less represented group. Five resampling methodologies have consequently undergone assessment: random oversampling, random undersampling, SMOTE (31), SMOTE-Tomek, and SMOTE-ENN (32). The most suitable approach was identified according to comprehensive forecasting effectiveness, with specific focus placed on balanced prediction accuracy and the Matthews correlation coefficient (MCC).

### 2.5 Model development

#### 2.5.1 Machine learning models

Data processing and model development were carried out using Python 3.10. Long Short-Term Memory (LSTM) and Gated Recurrent Unit (GRU) models were compared for temporal feature extraction. Mortality risk prediction was conducted using five classifiers: Logistic Regression (LR), Random Forest (RF), Light Gradient Boosting Machine (LightGBM), Extreme Gradient Boosting (XGBoost), and Multilayer Perceptron (MLP).

#### 2.5.2 Model training methods

The dataset was split into training and testing sets with a 7:3 ratio. Within the training set, 5-fold cross-validation with GridSearchCV was used for hyperparameter tuning. The final tuned models were evaluated on the held-out test set; for each of nine metrics (accuracy, precision, recall, F1 score, AUC, Balanced ACC, MCC, Brier score, and Youden's index), we report the point estimate with 95% bootstrap confidence intervals.

#### 2.5.3 Model interpretability

Feature importance was evaluated using Shapley Additive Explanations (SHAP), which assign an additive contribution value to each feature for an individual prediction. SHAP supports both global and local interpretation by quantifying the marginal influence of each feature on model output (33). To further enhance clinical interpretability, Local Interpretable Model-agnostic Explanations (LIME) was also applied (14, 34). By approximating the model's local decision boundary, LIME generates patient-specific feature weights that indicate how individual variables affect predicted mortality risk. Combining SHAP and LIME improved model transparency at both the population and individual-patient levels.

### 2.6 Dataset description

Baseline characteristics of categorical variables were summarized as frequencies and percentages, while continuous variables were reported as means ± standard deviations or medians (interquartile range), depending on distribution. Appropriate statistical tests, such as t-tests, chi-square tests, or non-parametric tests, were applied based on variable types and distributions. Statistical significance was set at *α* = 0.05.

## 3 Results

### 3.1 Baseline characteristics

Through the implementation of pre-specified inclusion and exclusion benchmarks, the final study population encompassed 4,205 individuals suffering from pneumonia, among whom 2,751 were sourced from the MIMIC-IV database (Data A) for the establishment of predictive models and 1,454 were obtained from the eICU cohort (Data B) for the performance of external validity assessment. Disorders affecting the circulatory and respiratory tracts were the leading admitting diagnoses, making up roughly half of all clinical cases across the two data collections. Within Data A, 46 out of 2,751 enrolled subjects (1.67%) experienced fatal outcomes during the dead 1 phase, 95 of 2,705 remaining participants (3.51%) succumbed to illness during the dead 2 phase, and 77 of 2,610 remaining participants (2.95%) passed away during the dead 3 phase. The aggregated 7-day fatality ratio reached 7.92% (218/2,751). For the Data B, 78 of 1,454 recruited patients (5.36%) suffered mortality events during the dead 1 phase, 55 of 1,376 surviving individuals (4.00%) encountered death during the dead 2 phase, and 37 of 1,321 remaining cases (2.80%) had fatal outcomes during the dead 3 phase. The accumulated 7-day death rate stood at 11.69% (170/1,454).

The baseline discrepancies of the two datasets at the dead 1 and dead 3 phases have been demonstrated in the contents of Table 1 and Supplementary Table S1, respectively.

**Table 1:** Comparison of various characteristics in the patient data (dead 1).

	Data A (*n* = 2,751)		*P* value	Data B (*n* = 1,454)		*P* value
	Alive (*n* = 2,705)	Dead (*n* = 46)		Alive (1,376)	Dead (*n* = 78)	
Age (age Age)	64.15 ± 16.31	65.35 ± 16.98	0.620	64.53 ± 15.94	69.22 ± 14.21	0.011
Gender			0.944			0.881
Male	1,543 (57.04%)	26 (56.52%)		782 (56.83%)	45 (57.69%)	
Female	1,162 (42.96%)	20 (43.48%)		594 (43.17%)	33 (42.31%)	
Temperature	36.92 (36.63, 37.31)	36.70 (36.27, 37.18)	0.004	37.06 (36.87-37.27)	37.04 (36.59–37.43)	0.791
Heart rate	90.64 ± 16.88	94.91 ± 19.60	0.149	86.66 ± 16.05	103.06 ± 17.66	<0.001
Systolic Blood Pressure	115.09 ± 15.68	107.93 ± 14.05	0.001	123.10 ± 19.60	104.79 ± 17.04	<0.001
Diastolic blood pressure	63.46 ± 11.71	65.08 ± 11.59	0.351	66.59 ± 12.16	56.32 ± 11.64	<0.001
SO2	96.48 ± 2.5	95.42 ± 3.14	0.029	95.67 (93.67, 97.53)	93.41 (90.40, 95.90)	<0.001
Breathing	20.68 (17.93, 23.92)	21.27 (19.03, 25.28)	0.175	19.28 (17.86, 22.90)	23.35 (19.15, 27.29)	<0.001
Coronary disease	711 (26.28%)	11 (23.91%)	0.717	233 (16.93%)	10 (12.82%)	0.344
Diabetes	925 (34.20%)	10 (21.74%)	0.077	242 (17.59%)	14 (17.95%)	0.935
Hypertension	1,902 (70.31%)	25 (54.35%)	0.019	232 (16.86%)	8 (10.26%)	0.132
Lung cancer	163 (6.03%)	3 (6.52%)	0.889	33 (2.40%)	2 (2.56%)	0.926
Activated partial thromboplastin time	34.77 (29.30–47.41)	35.78 (29.80–52.33)	0.150	71.53 ± 55.29	80.45 ± 57.59	0.186
Alanine aminotransferase	30.50 (17.00, 70.00)	30.50 (19.38, 92.25)	0.452	48.00 (25.00, 155.38)	113.50 (43.00, 592.50)	<0.001
Albumin	2.95 ± 0.60	2.70 ± 0.63	0.010	2.60 (2.00, 3.20)	2.15 (1.90, 2.80)	0.003
Aspartate aminotransferase	46.00 (25.00, 111.00)	61.50 (31.25, 163.38)	0.141	57.00 (24.00, 321.00)	233.00 (85.75, 844.00)	<0.001
Blood sugar	131.00 (107.00, 168.50)	130.00 (100.81, 162.04)	0.313	124.78 (103.15, 156.69)	138.00 (106.19, 153.86)	0.181
Calcium	8.17 ± 0.86	8.17 ± 1.00	0.958	8.50 (7.90, 9.00)	7.10 (6.01, 7.90)	<0.001
Chlorine	103.38 ± 7.17	101.70 ± 7.72	0.150	103.61 ± 6.51	105.62 ± 7.98	0.032
Creatinine	1.15 (0.77, 1.95)	1.38 (0.88, 2.04)	0.587	0.96 (0.66, 1.70)	2.07 (1.12, 3.10)	<0.001
Hemoglobin	10.18 ± 2.10	10.38 ± 2.27	0.559	9.60 (8.40, 11.20)	9.20 (7.81, 10.83)	0.134
International normalized	1.35 (1.20, 1.70)	1.51 (1.30, 2.02)	0.008	1.50 (1.13, 2.35)	2.42 (1.70, 3.12)	<0.001
Lactate dehydrogenase	309.00 (229.00, 481.00)	416.25 (265.00, 747.25)	0.006	364.00 (193.00, 495.25)	203.00 (145.00, 527.00)	0.330
Platelet	179.00 (110.50, 254.00)	152.62 (95.08, 261.25)	0.338	232.00 (153.75, 318.25)	137.50 (76.00, 200.50)	<0.001
Potassium	4.21 ± 0.62	4.42 ± 0.67	0.040	3.90 (3.60, 4.30)	4.27 (3.70, 5.10)	<0.001
Red blood cell	3.41 ± 0.75	3.53 ± 0.77	0.313	3.41 ± 0.69	3.31 ± 0.75	0.270
Sodium	138.24 ± 5.79	137.45 ± 5.90	0.371	139.00 (136.00, 142.00)	139.75 (135.08, 142.88)	0.699
Total bilirubin	0.70 (0.40, 1.60)	0.68 (0.43, 2.27)	0.321	0.70 (0.30, 1.60)	1.50 (0.62, 3.20)	<0.001
White blood cell	11.65 (7.90, 16.58)	14.12 (11.37, 17.82)	0.015	10.10 (7.60, 14.01)	15.27 (9.87, 28.37)	<0.001
Respiratory type	2,518 (93.09%)	46 (100%)	0.072	1,326 (96.37%)	76 (97.44%)	1.000

### 3.2 Model selection

**Figure 2:**
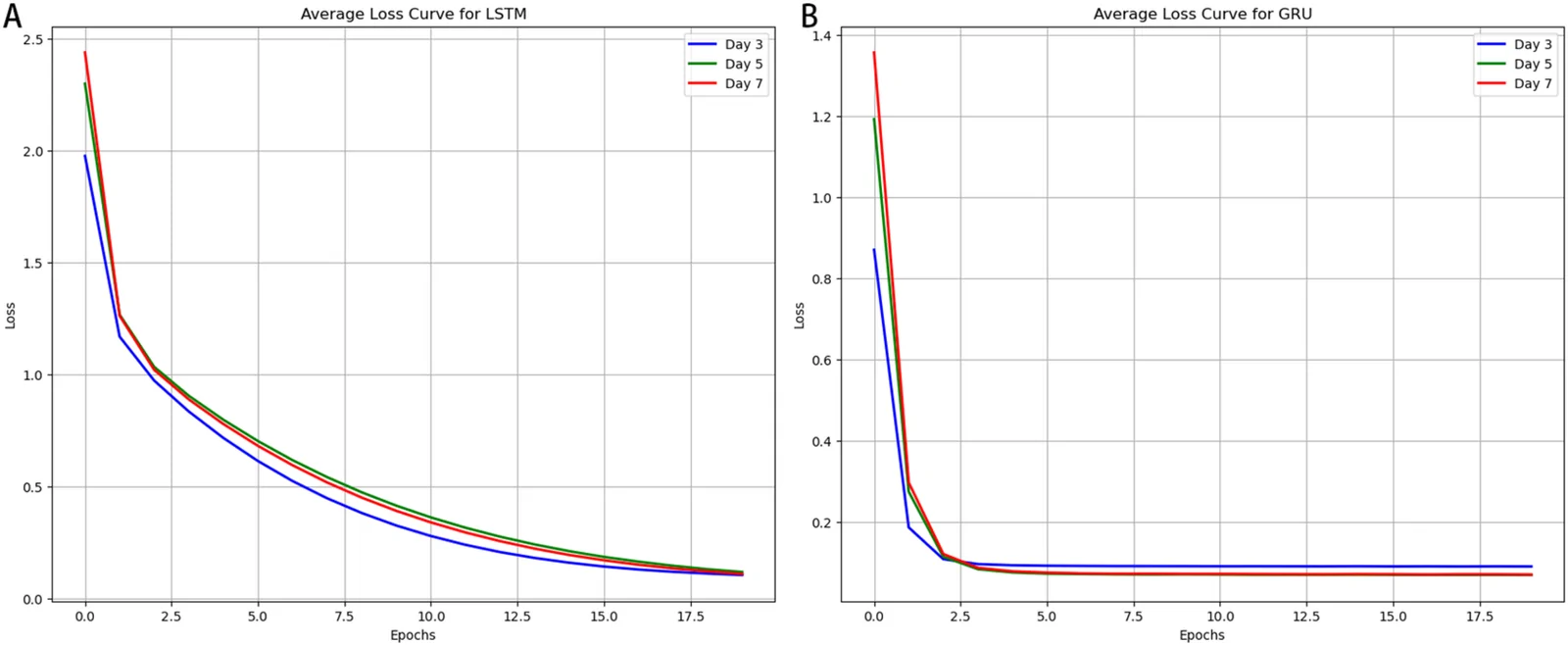
Training loss curves for the model across different prediction tasks.

As presented in Table 2, SMOTE attained the foremost comprehensive performance across the five resampling approaches, and thus was chosen for follow-up model training and assessment. Following the completion of data balancing, separate evaluations of LSTM and GRU for temporal feature extraction have been conducted (Figure 2). The GRU framework achieved convergence at a fast pace during the initial phase of training, with its loss value dropping drastically from 2.4 to nearly 0.1 after roughly 2.5 iterations, which reveals the high efficiency of feature capture within a brief time frame. Nevertheless, the loss entered a plateau phase afterwards, which imposed restrictions on further enhancement of model performance. In comparison, even though the LSTM framework underwent slower optimization at the outset, its loss kept decreasing and eventually converged to a smaller numerical value as the training process advanced, especially in assignments that demand the grasping of long-range interdependencies. The LSTM framework was accordingly picked for the task of temporal feature extraction.

**Table 2:** Regression index of different sampling methods (dead 3).

Resampling Algorithms	ROC-AUC	PR-AUC	Recall	Balanced ACC	MCC	Threshold
Raw data	0.883	0.610	0.523	0.749	0.555	0.5
Random Over-Sampling	0.878	0.655	0.455	0.720	0.553	0.5
Under sampling	0.715	0.212	0.682	0.683	0.208	0.5
SMOTE	0.857	0.620	0.568	0.774	0.610	0.5
SMOTE-Tomek	0.840	0.600	0.477	0.725	0.500	0.5
SMOTE-ENN	0.765	0.425	0.568	0.724	0.335	0.5

The long short-term memory computation framework has taken delivery of a three-dimensional input matrix encompassing the quantity of samples, time steps, and clinical characteristic indices. The entire neural network is made up of an LSTM layer with 64 processing units, which is followed by a dropout layer featuring a dropout proportion of 0.20, a fully connected dense layer equipped with 32 neurons to produce the hidden temporal feature representation, a second dropout layer, and a sigmoid output layer. Focal loss was employed in the course of model training to alleviate the impacts of category imbalance. The 32-dimensional hidden features procured via the LSTM module were subsequently utilized as input values for downstream classification algorithms, which encompass LR, RF, XGBoost, LightGBM, and MLP.

### 3.3 Model prediction performance

**Figure 3:**
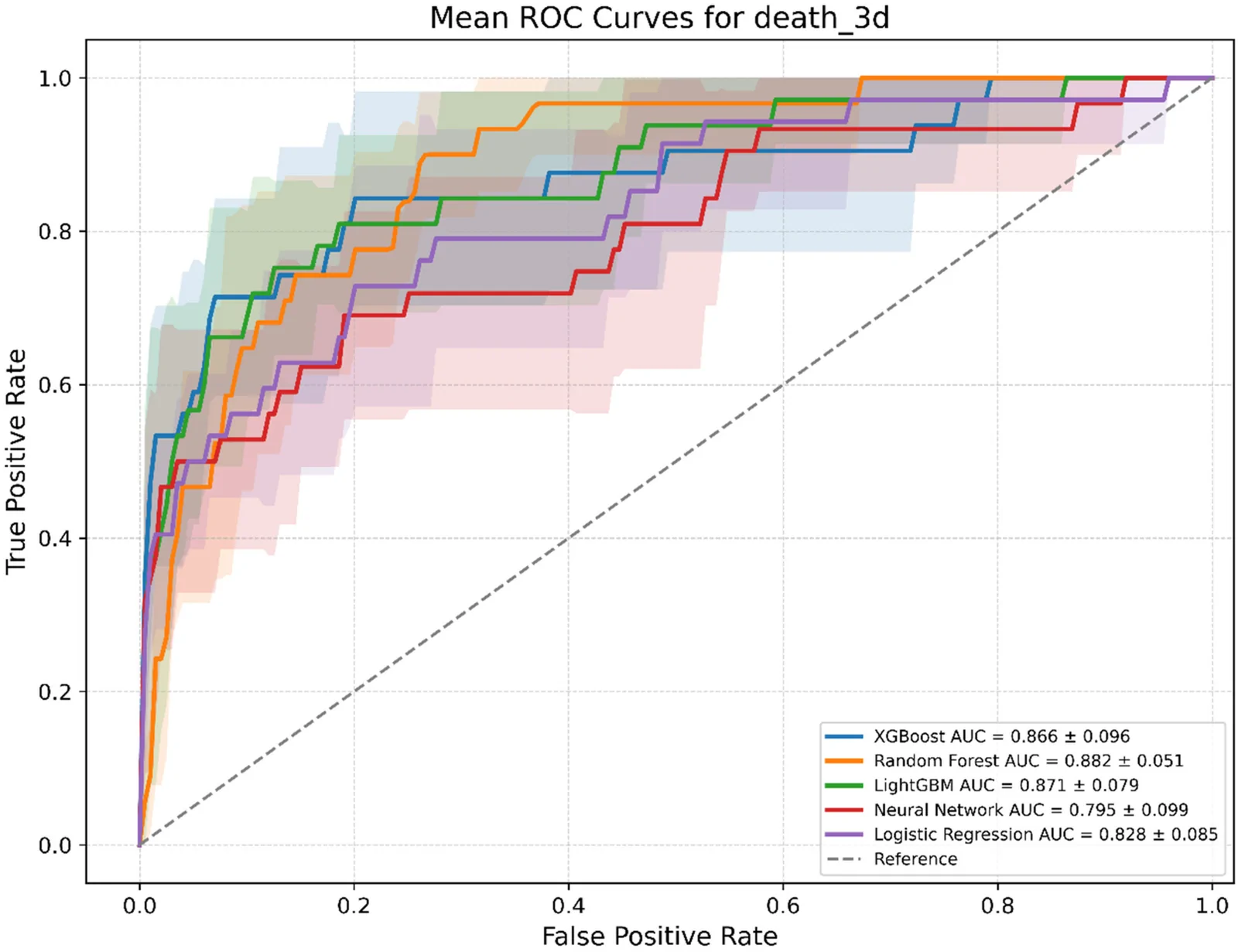
Five-fold cross-validation ROC curves for five learning models.

In this study, GridSearchCV was employed to optimize specific hyperparameters of the model. For detailed information, refer to the supplementary document (Supplementary Table S4). Table 3 presents the model performance and its 95% CIs from 5-fold cross-validation. As shown in Table 3, each model demonstrates distinct advantages in target learning tasks. Regarding individual model performance, the RF model showed the best overall performance, achieving the highest accuracy, precision, Balanced ACC, MCC and F1 score, together with the lowest Brier score. In contrast, the LR model achieved the highest recall, AUC, and Youden's index, indicating strong sensitivity and discrimination. The AUC visualization for 5-fold cross-validation is shown in Figure 3. Model performance during the dead 1 and dead 2 stages is summarized in Supplementary Tables S2 and S3, respectively.

**Table 3:** Predictive performance of machine learning models (dead 3).

Measure	LR	RF	LightGBM	XGBoost	MLP	**External validation**
Accuracy	0.956 (0.948–0.963)	0.961 (0.954–0.969)	0.955 (0.946–0.963)	0.957 (0.953–0.961)	0.957 (0.948–0.965)	0.859 (0.842–0.876)
Precision	0.856 (0.751–0.961)	0.938 (0.853–1.000)	0.856 (0.767–0.946)	0.882 (0.805–0.960)	0.875 (0.785–0.965)	0.437 (0.379–0.495)
Recall	0.843 (0.771–0.916)	0.823 (0.735–0.912)	0.823 (0.735–0.912)	0.830 (0.739–0.922)	0.830 (0.739–0.922)	0.711 (0.640–0.777)
F1 Score	0.846 (0.800–0.893)	0.874 (0.824–0.924)	0.837 (0.782–0.892)	0.852 (0.820–0.883)	0.849 (0.792–0.906)	0.541 (0.483–0.594)
AUC	0.947 (0.901–0.993)	0.940 (0.904–0.975)	0.944 (0.904–0.984)	0.940 (0.902–0.979)	0.944 (0.894–0.993)	0.826 (0.790–0.862)
Balanced ACC	0.910 (0.876–0.944)	0.914 (0.872–0.957)	0.904 (0.871–0.937)	0.911 (0.862–0.960)	0.904 (0.863–0.945)	0.798 (0.762–0.833)
MCC	0.812 (0.759–0.864)	0.862 (0.831–0.893)	0.841 (0.816–0.866)	0.855 (0.820–0.889)	0.841 (0.814–0.867)	0.488 (0.427–0.547)
Brier Score	0.024	0.020	0.025	0.023	0.023	0.070
Youden's index	0.830	0.818	0.811	0.820	0.819	0.590

**Figure 4:**
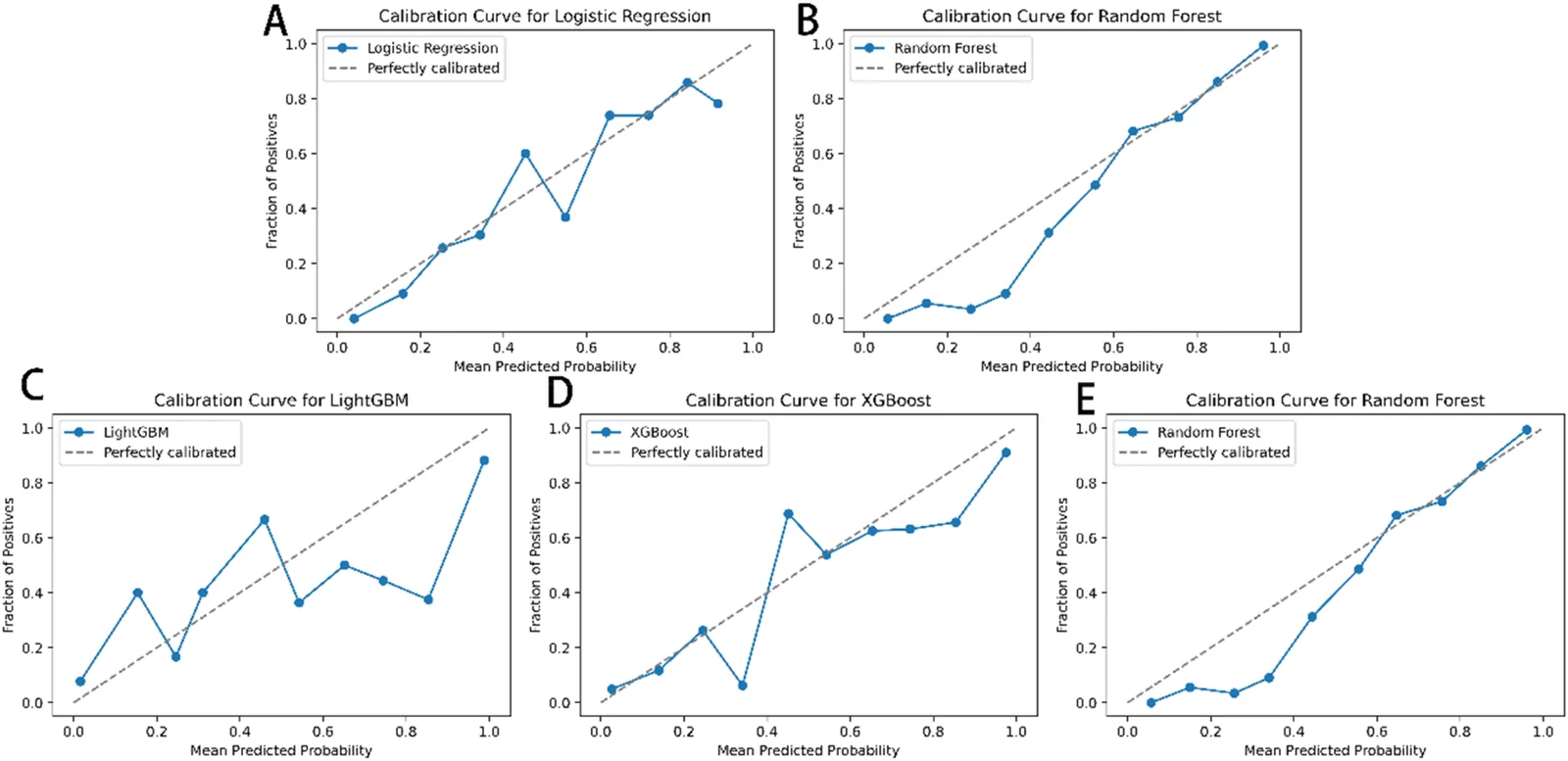
Calibration curves for five models. **(A)** Logistic regression, **(B)** Random forest, **(C)** Light Gradient Boosting Machine, **(D)** Extreme Gradient Boosting, **(E)** Neural network.

**Figure 5:**
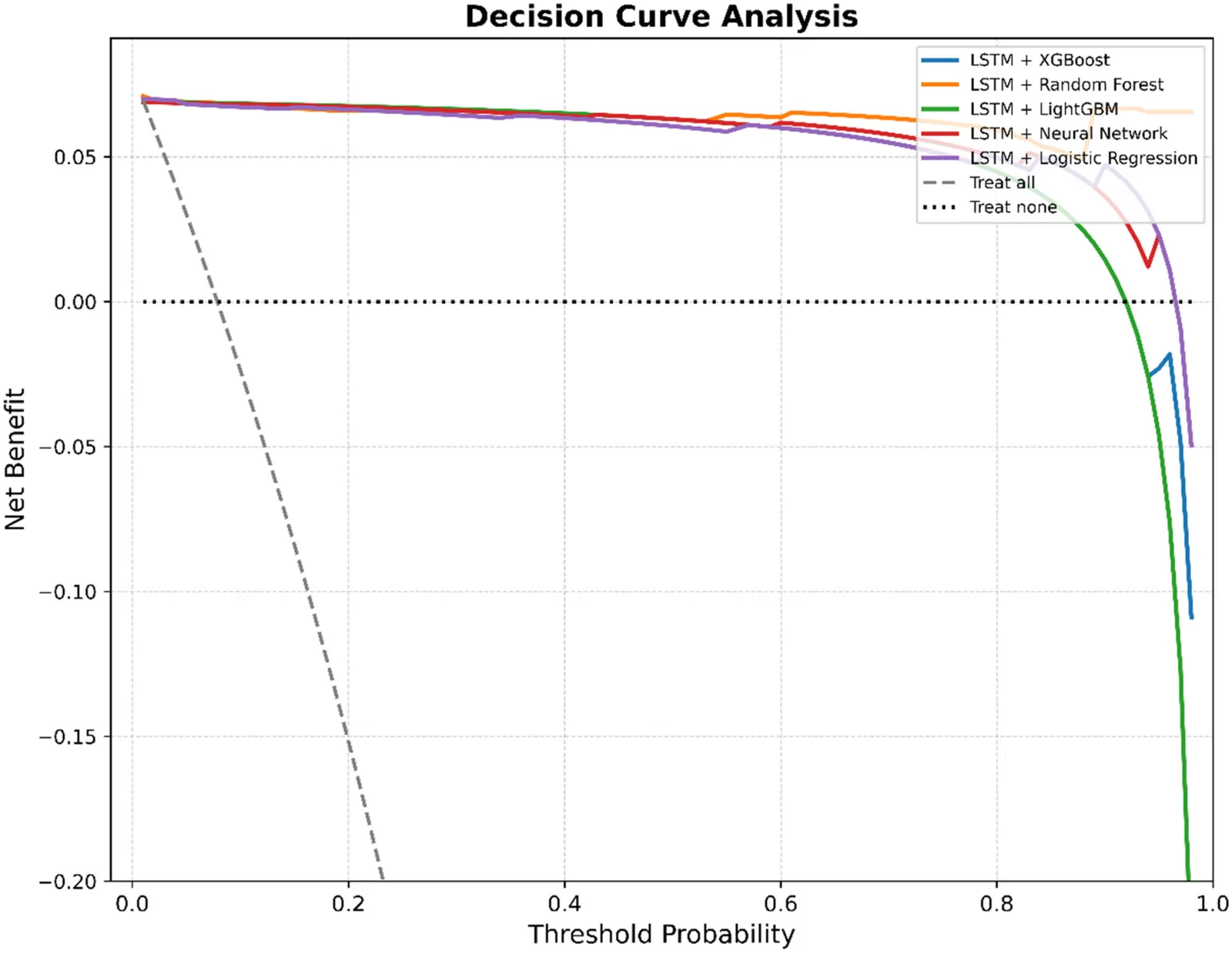
DCA curve.

Illustrated in Figure 4 are the calibration curves pertaining to all constructed prediction models. RF, LightGBM, and XGBoost demonstrated outstanding discrimination capability, as mirrored by the values of their corresponding calibration slopes documented in Table 3. Nevertheless, the decision curves of LightGBM and XGBoost fluctuate significantly. Further assessment of decision curve analysis outcomes, as displayed in Figure 5, uncovered that RF attained the maximum net benefit and maintained a continuously positive value throughout the entire assessed threshold scope. In aggregate, RF exhibited reliable performance across a set of multiple evaluation indicators, and was consequently chosen as the optimal model for follow-up analysis and result interpretation.

### 3.4 Model interpretability

**Figure 6:**
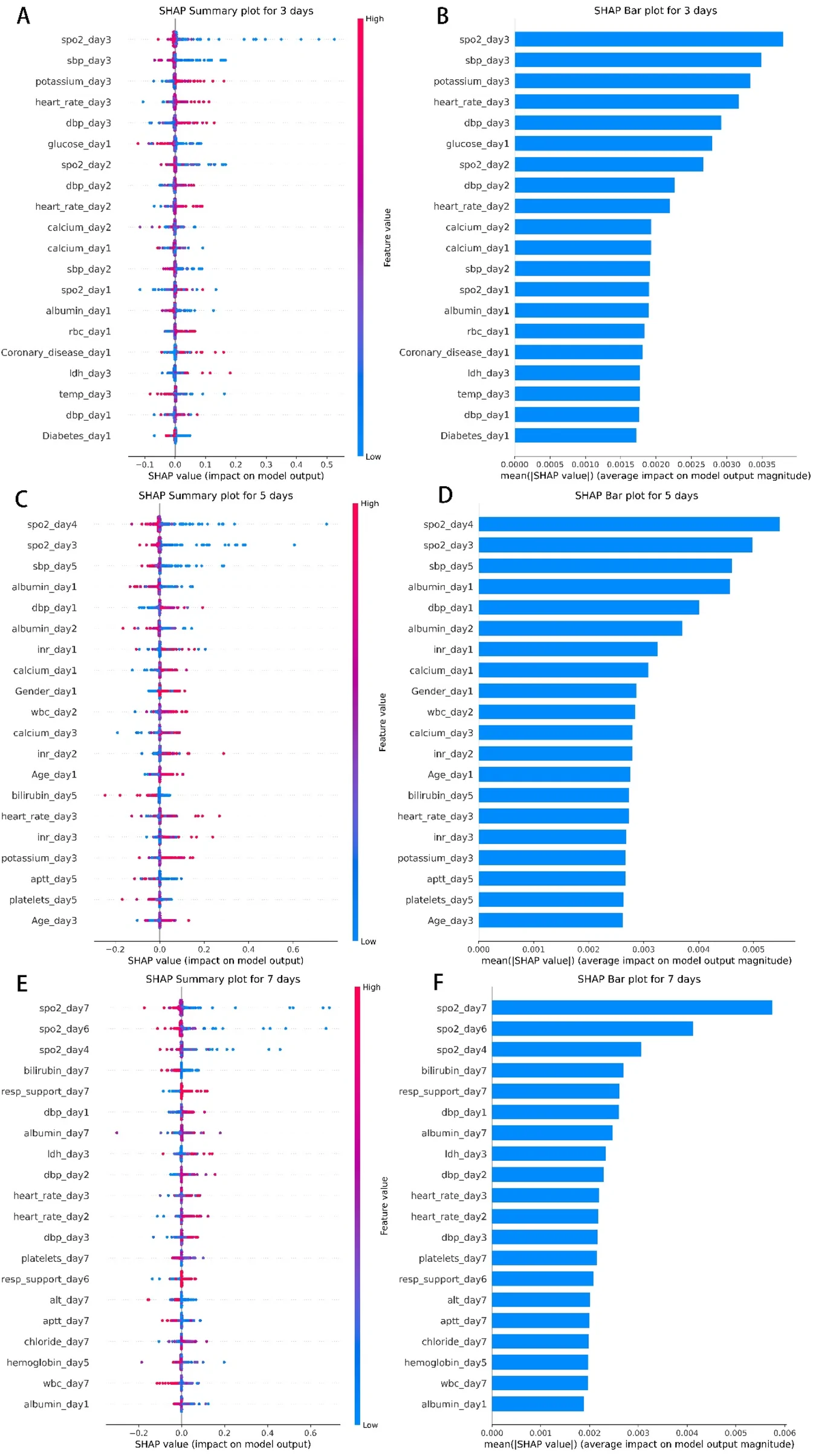
SHAP variable importance rankings based on the random forest model. **(A)** and **(B)** : SHAP graph at “dead 1”, **(C)** and **(D)** : SHAP graph at “dead 2”, **(E)** and **(F)** : SHAP graph at “dead 3”. **(A, C)**, and **(E)** show SHAP summary plots for the 3-day, 5-day, and 7-day mortality prediction models, respectively. Each dot represents an individual patient, and the position on the *x*-axis indicates the direction and magnitude of the feature contribution to the model output. Red represents higher feature values, while blue represents lower feature values. **(B, D)**, and **(F)** show the corresponding SHAP bar plots, ranking variables according to the mean absolute SHAP value.

We have conducted the implementation of SHAP analytical approaches on the finalized LSTM–RF model for the evaluation of the significance of features across three distinct time windows (Figures 6A–F). The dynamic tracking of core variables, which encompass oxygenation-associated markers, albumin, and respiratory support measures, has uncovered the temporal variations of feature significance that may lend support to the delineation of fluctuations in the risk of short-term mortality.

**Figure 7:**
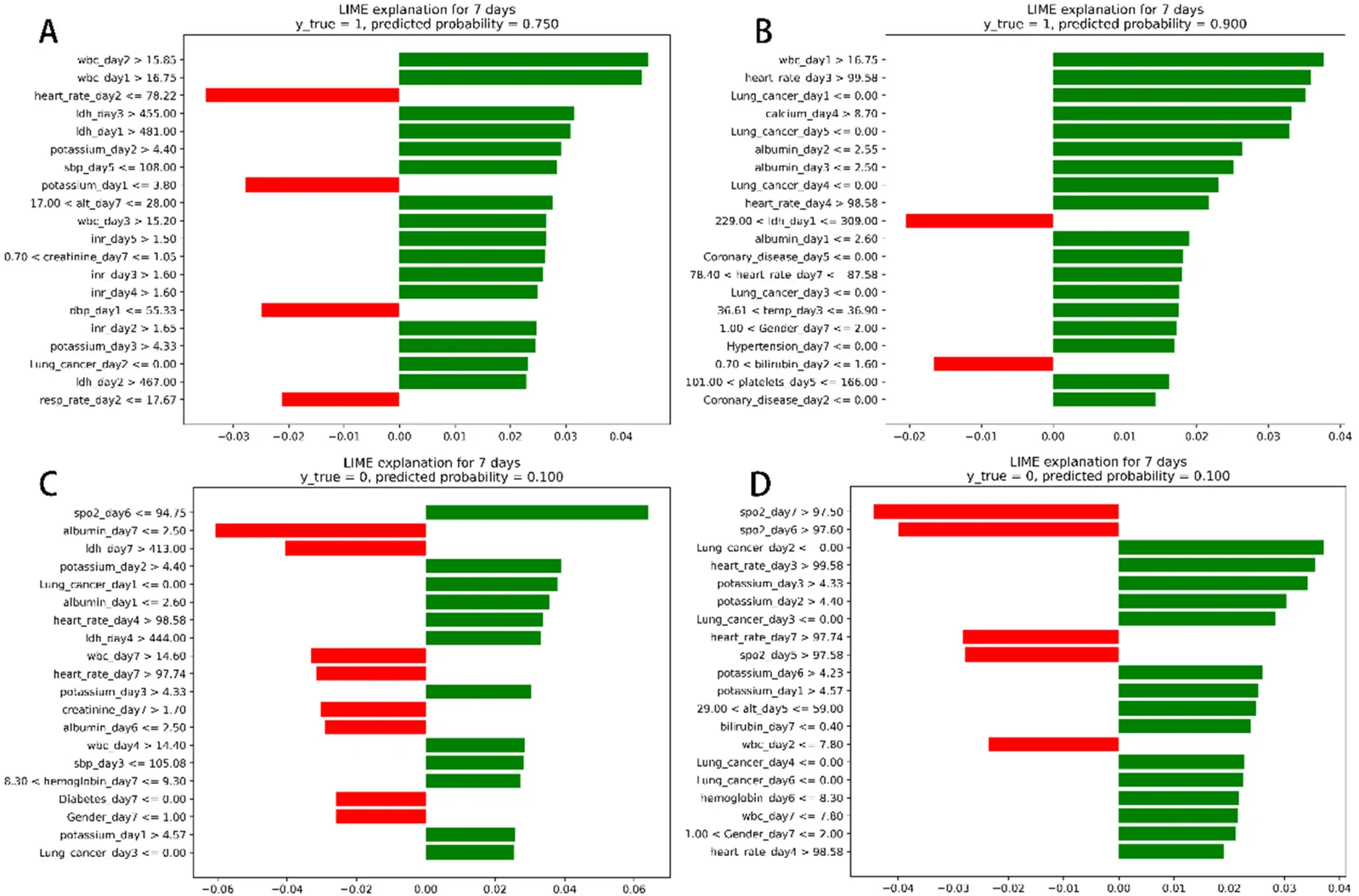
LIME locally interpretable model agnostic interpretation map. This graph is based on the “dead 3” stage of the RF. **(A)** and **(B)** serve as representations of true-positive instances with elevated predicted mortality probability levels, while **(C)** and **(D)** stand for true-negative cases with comparatively low death risk forecasting values. Green-colored bars denote characterizing factors that have brought about an increase of the model's predictive likelihood for the target category, and red-colored bars signify marking attributes that have induced a reduction of the aforementioned metric.

LIME analysis was further conducted to provide individualized explanations for representative predictions generated by the final LSTM–RF model (Figure 7). Four representative cases were selected based on the confusion matrix: two true-positive cases and two true-negative cases. Figures A and B depict true-positive cases, in which patients died within 7 days and were correctly assigned high predicted mortality probabilities. Figures C and D depict true-negative cases, in which patients survived and were correctly assigned low predicted mortality probabilities.

In the two true-positive examples, the model predictions were mainly supported by variables reflecting systemic inflammation, organ dysfunction, and physiological instability. For example, elevated white blood cell count, increased lactate dehydrogenase, abnormal coagulation-related indicators such as INR, electrolyte abnormalities, impaired renal function, and abnormal vital signs contributed positively to the predicted mortality risk. In contrast, the two true-negative cases showed low predicted mortality probabilities. In these patients, several features contributed to suppressing the predicted risk, including relatively preserved oxygenation-related variables, lower inflammatory or organ-injury indicators, and more stable physiological profiles. Although some variables still increased the predicted risk locally, their contribution was insufficient to shift the overall prediction toward death. This indicates that the model was able to distinguish lower-risk survivors by integrating multiple longitudinal clinical features rather than relying on a single variable. Overall, these representative LIME explanations suggest that the LSTM–RF model provided clinically interpretable individualized predictions.

### 3.5 External validation

An overall count of 1,454 ICU patients suffering from pneumonia within the eICU cohort have been encompassed in the external verification process. The 7-day in-hospital fatality ratio reached 11.69%, which is elevated in comparison with the 7.92% identified in the MIMIC-IV dataset. Prior to the conduct of verification, the eICU data were subjected to the identical preprocessing workflows as the MIMIC-IV dataset, which encompass feature standardization to compensate for discrepancies in the distribution pattern of variables. Within the independent participant group, the random forest framework has maintained favorable predictive capability, with an accuracy value of 0.859, an AUC of 0.826, a Brier score of 0.070, and a Youden's index of 0.590. Predictive efficacy showed a reduction in comparison with the internal verification process, especially for precision level, which experienced a drop from 0.938 to 0.437, suggesting the presence of more false positive forecasting outcomes in the external data collection. Even so, the recall metric stayed at 0.711, revealing that the predictive framework still recognized a considerable share of patients with elevated risk status. In general, the RF model has displayed a specific extent of transferability to different cohorts.

## 4 Discussion

Although mortality rates among ICU patients with pneumonia have been reported to be approximately 25%–30% (35, 36), the 7-day mortality rate in the present study was lower, at 7.92% in Data A and 11.69% in Data B, respectively. This difference may be partly explained by the reverse time-window design used in our study. Rather than predicting mortality from a fixed baseline, such as ICU admission, we extracted clinical information from the seven days preceding death or discharge. Patients with very early death or insufficient longitudinal data were also excluded. As a result, the final cohort consisted of patients with available dynamic observations before the outcome event, rather than all ICU pneumonia patients at admission. This may have reduced the proportion of rapid early deaths and contributed to the lower observed mortality rate, while still allowing the model to identify temporal risk patterns before clinical deterioration.

As the reverse time-window study design produced a comparatively small share of mortality occurrences in the final study cohort, class imbalance constituted a critical methodological obstacle. For the purpose of tackling this concern, we have formulated an explainable dynamic framework for the predicting 7-day mortality risk among ICU patients suffering from pneumonia, and have conducted a systematic comparison of multiple resampling strategies, encompassing random oversampling, random undersampling, SMOTE, and hybrid approaches. SMOTE attained the best overall performance and was hence chosen for follow-up model training activities. In contrast to naive oversampling, SMOTE creates artificial minority-class samples inside the feature space of existing minority-class samples instead of only replicating original instances. This methodology boosted minority-class representation, reduced the model's bias toward the majority class, and strengthened the detection of high-risk patients, lending support to its application in imbalanced clinical prediction tasks (37).

In model development, a hybrid RF model enriched with LSTM features was proposed to capture dynamic characteristics and complex nonlinear relationships within time-series data. The model leveraged LSTM's ability to extract multi-timepoint latent features, combined with RF's strong classification capability, to deliver strong predictive performance (38, 39). On the internal test dataset, the model achieved an AUC of 0.940 (95% CI: 0.904–0.975) outperforming other machine learning approaches. To assess the model's generalizability and stability, independent external validation was performed using the eICU. The external validation results provided further evidence of its generalizability.

The “black-box” characteristic of machine learning frameworks frequently complicates the comprehension of their predictive outputs, raising worries regarding the safety of patients and restricting clinical implementation (40). To tackle these problems, we evaluated model interpretability using SHAP and LIME.

SHAP analysis revealed distinguishable time-related patterns within the contributing elements of mortality hazard across the “dead 1”, “dead 2”, and “dead 3” prediction windows. For the “dead 1” time frame, the variables with the most salient impact were chiefly acute physiological markers with close proximity to the endpoint occurrence, encompassing SpO₂, systolic blood pressure, potassium, heart rate, and diastolic blood pressure. This observed outcome implies that short-term death risk was principally triggered by acute cardiorespiratory and metabolic instability instead of baseline features in isolation. Of particular note, reduced lower SpO₂ was consistently associated with higher predicted mortality risk, showing that impaired oxygenation was a core indicator of impending clinical worsening. In ICU cases suffering from pneumonia, deteriorating gas exchange may arise from alveolar inflammation, ventilation-perfusion mismatch, pulmonary edema, or advancement toward respiratory breakdown (41, 42). When compensatory biological pathways are exceeded, hypoxemia can quickly worsen tissue oxygen deficit, circulatory unsteadiness, and metabolic disruption, which may account for why oxygenation and hemodynamic parameters held a dominant position in the “dead 1” analytical model.

In the “dead 2” window, oxygenation-related variables remained dominant, with SpO₂ at days 3 and 4 showing the highest feature importance. However, additional variables, including albumin, INR, calcium, WBC, bilirubin, and blood pressure, also became prominent. This pattern suggests that the “dead 2” window captures not only acute respiratory deterioration but also the patient's underlying physiological reserve and early systemic dysfunction. During this intermediate phase, persistent pulmonary infection and hypoxemia may trigger a systemic inflammatory response, leading to endothelial activation, increased vascular permeability, impaired microcirculation, and progressive organ stress (43, 44). Therefore, variables related to inflammation, nutritional status, coagulation, metabolism, and liver function begin to gain predictive importance. Albumin may reflect nutritional reserve, inflammatory burden, and capillary leakage, while INR and bilirubin may indicate early coagulation and hepatic involvement (45, 46). These changes suggest that the “dead 2” model identifies a transitional stage in which local pulmonary injury begins to evolve into systemic physiological derangement.

The transition from “dead 1” to “dead 2” may therefore represent a critical phase in disease progression. Compared with the “dead 1” model, which was mainly characterized by acute changes in oxygenation, blood pressure, heart rate, and potassium, the “dead 2” model incorporated broader indicators of systemic deterioration. This shift implies that mortality risk during this period may transition from being primarily driven by immediate physiological instability to being influenced by evolving inflammatory, metabolic, nutritional, and coagulation-related abnormalities.

For the “dead 3” prediction window, the SHAP results showed a broader and more complex pattern of feature importance. SpO₂ remained the most important predictor, particularly at days 7, 6, and 4, confirming that persistent or progressive oxygenation impairment is a stable signal of poor prognosis. In addition, respiratory support at days 6 and 7 became one of the leading predictors, suggesting that the need for advanced or sustained respiratory support reflects increasing disease severity and reduced pulmonary reserve. As the disease progresses, patients who remain dependent on respiratory support may have persistent alveolar injury, severe ventilation-perfusion mismatch, respiratory muscle fatigue, or evolving acute respiratory distress. Thus, respiratory support is not only a treatment-related variable but also a surrogate marker of the severity and persistence of respiratory failure (47).

Within the “dead 3” prediction framework, variables associated with organ damage and systemic functional impairment, encompassing bilirubin, albumin, LDH, platelets, ALT, APTT, chloride, and WBC, were also identified as foremost predictive factors. This finding demonstrates that extended observation timeframes permit the framework to capture the cumulative effects of multi-organ functional abnormality. In this stage of disease progression, the pathological cascade may have spread beyond restricted pulmonary infection. Sustained inflammatory responses and tissue oxygen deficiency can facilitate endothelial damage, coagulation triggering, mitochondrial dysfunction, and microthrombus generation, leading to compromised oxygen transportation at the tissue level notwithstanding supportive interventions (48). Coagulation dysregulation and platelet parameter alterations may mirror inflammation-triggered hemostatic disequilibrium and endothelial functional damage, while hepatic function markers and LDH may reflect systemic tissue impairment, metabolic strain, and organ hypoperfusion (49, 50). As a result, the “dead 3” prediction framework may reflect a more progressed phase of disease advancement, typified by respiratory failure coupled with systemic inflammation, coagulation dysregulation, metabolic imbalance, and multi-organ damage.

Overall, SHAP analysis suggested a transition from acute cardiorespiratory instability to broader systemic organ dysfunction as the prediction window expanded. This temporal pattern supports dynamic monitoring of oxygenation status, hemodynamic stability, coagulation function, inflammatory markers, and organ function in ICU patients with pneumonia.

LIME further complemented SHAP by providing individualized explanations for specific predictions. In correctly predicted high-risk cases, LIME showed that lower SpO_2_, elevated WBC, increased heart rate, abnormal LDH, reduced albumin, and changes in calcium or blood pressure increased the predicted mortality risk. These case-level findings were generally consistent with the global SHAP results, suggesting that the model's predictions were driven by clinically meaningful patterns involving respiratory deterioration, inflammatory response, cardiovascular stress, and metabolic or nutritional disturbance. In contrast, relatively preserved physiological indicators or the absence of specific comorbidities reduced the predicted risk in some individuals. Thus, the combined use of SHAP and LIME improves model transparency by linking population-level risk patterns with patient-specific individual-level prediction patterns, supporting individualized risk assessment and clinical decision-making.

In the external validation cohort, the model maintained acceptable discrimination, but precision decreased from 0.938 in the internal test cohort to 0.437 in the eICU cohort, indicating a substantial reduction in positive predictive value. This suggests that although the model retained some ability to distinguish higher-risk from lower-risk patients, its positive predictive value was reduced when applied to an independent dataset.

This decline may be partly explained by heterogeneity between MIMIC-IV and eICU. The two databases differ in patient populations, institutional settings, coding systems, measurement frequency, and data-recording processes. Several representative variables, including blood pressure, oxygen saturation, respiratory rate, creatinine, INR, platelet count, and WBC, showed different distributions or mortality-related patterns across cohorts. In addition, reference ranges, unit information, and variable definitions were not always consistently available or directly comparable in eICU. These cross-database differences may have shifted the predicted risk distribution and increased false-positive predictions in the external cohort.

These uncovered observations also underscore the requirement for prudence when transferring the put forward analytical structure into real-world clinical application. As time-dependent parameters were aligned in a retrospective manner relative to patient mortality or hospital discharge, the currently established model chiefly recognizes dynamic patterns linked to short-term fatality risk instead of directly imitating real-time prognostic estimation during intensive care unit admission. When implemented in future clinical settings, this prediction model could be modified into a continuous prognostic framework and administered every 12–24 h. Through integration of daily refreshed clinical data, encompassing vital signs, laboratory testing outcomes, and therapy-related parameters, the computational tool could generate repeated short-term mortality risk assessments over the course of treatment. The generated result could be presented as both the existing risk value and its changing tendency across time, enabling clinical practitioners to monitor if a patient's risk level is ascending, descending, or staying unchanged. Through this mechanism, the prognostic model could facilitate dynamic risk categorization throughout ICU admission. Patients with continuously elevated or fast rising projected risk could be marked for more intensive surveillance, re-evaluation of therapeutic regimens, or cross-departmental assessment. Nevertheless, these prognostic estimations should be construed as auxiliary reference material and ought not to substitute clinical decision-making capacity or be utilized as an independent decision-making instrument.

Most previous studies on pneumonia mortality prediction have framed the task as binary classification, and relatively fewer have focused specifically on ICU patients with pneumonia. For example, Kang et al. (51) reported an AUC of 0.844 for 30-day mortality prediction in pneumonia patients, Lee et al. (52) reported that the best-performing model achieved an accuracy of 0.747 in ICU pneumonia patients, and Jeon et al. (12) compared LR, gradient boosting decision trees, and MLP models for ICU pneumonia mortality prediction, and reported that the MLP model achieved an accuracy of 0.822, precision of 0.860, recall of 0.440, and AUC of 0.856.

Beyond pneumonia-specific prediction studies, previous work has also applied advanced deep learning-based time-series models to ICU mortality prediction using longitudinal EHR data. For example, RNN-, GRU-, and LSTM-based models in MIMIC-IV have achieved AUC values of approximately 0.86–0.87 (53), while recurrent neural network models using heterogeneous MIMIC-III ICU time-series data have reported an AUROC of approximately 0.85 after 48 h (54). Attention-based temporal convolutional networks have also shown acceptable performance for ICU mortality prediction, with reported AUCROC values around 0.84 (55). These studies generally reported acceptable discrimination, with performance varying according to cohort characteristics, prediction windows, input features, and validation strategies. The performance of our LSTM-RF model was broadly comparable to these previous deep learning-based ICU mortality prediction studies.

## 5 Limitations

This study has several limitations. First, as a retrospective study based on MIMIC-IV and the eICU database, the results may be affected by data quality, missingness, and differences in data-recording practices. Some clinically important variables, including detailed ventilator parameters, pathogen information, antimicrobial treatment, and other treatment-related data, were unavailable or incompletely recorded, which may have led to residual confounding. Second, although missing values were handled using forward filling and multiple imputation, these methods rely on assumptions about the missing-data mechanism. Forward filling may smooth abrupt physiological changes, while imputation cannot fully recover unmeasured information. Because missingness in ICU databases may be related to disease severity, monitoring intensity, or clinical decision-making, imputed values may still introduce uncertainty and affect generalizability. Third, class imbalance was addressed using SMOTE in the training set. Although this approach may improve the recognition of mortality events, synthetic minority-class samples may not fully represent real ICU patients and may influence calibration and threshold-dependent metrics, such as precision, recall, and positive predictive value.

Fourth, several preprocessing and modeling choices may limit interpretation. Daily mean aggregation improved consistency across databases but may have reduced within-day temporal information, such as transient hypoxemia or abrupt hemodynamic instability. In addition, the LSTM component generated latent temporal representations, which are less directly interpretable. Although SHAP and LIME were used to improve transparency, these *post hoc* explanations may not fully reflect the internal decision process of the model. Finally, the retrospective 7-day window before death or discharge does not fully represent a prospective prediction setting. Variables measured close to death may reflect advanced deterioration, treatment escalation, or end-of-life care. Although external validation was performed, the reduced precision in the eICU cohort suggests that prospective multicenter validation, local recalibration, real-time EHR integration, and clinical impact assessment are needed before clinical implementation.

## 6 Conclusion

Pneumonia remains a major cause of mortality among ICU patients, and timely recognition of factors associated with clinical deterioration is of critical importance for risk surveillance and prompt therapeutic action. In this study, we developed an interpretable LSTM–RF framework using data from two independent databases to assess short-term mortality risk across three dynamic time windows within the seven days preceding the outcome event. Through integration of serial clinical data, the prediction model attained satisfactory prognostic accuracy and uncovered time-dependent variations in core elements linked to mortality risk. External verification also demonstrated reasonable generalizability across heterogeneous patient cohorts. External validation also indicated a certain degree of generalizability across different patient populations.

This dynamic framework may help identify critical transition periods during disease progression and support time-sensitive risk assessment in ICU patients with pneumonia. Interpretability analyses further enhanced model transparency by linking population-level risk patterns with patient-specific prediction mechanisms. Nevertheless, the framework should be regarded as a retrospective dynamic risk assessment model rather than a clinically deployable real-time decision support tool. Prospective multicenter validation, real-time EHR integration, local recalibration, and workflow-based evaluation are required before clinical implementation.

## Data Availability

The data analyzed in this study is subject to the following licenses/restrictions: This data can be obtained from the following website: https://physionet.org/content/mimiciv/3.1/. Requests to access these datasets should be directed to Yihai zhai, zhaizzzxxx2021@163.com.
