# Supplementary material for: Time-Series modeling for predicting mortality risk in intensive care unit patients with pulmonary inflammation
Source: Front Med (Lausanne). 2026 Sep 3;13:1852032. doi: 10.3389/fmed.2026.1852032 (PMC13581569; doi:10.3389/fmed.2026.1852032)
Supplement: Supplementary file 1 [file Table1.pdf]

Title: Time-Series Modeling for Predicting Mortality Risk in intensive care unit Patients with Pulmonary Inflammation

Author: Yihai Zhai; Ruixin Xu; Haisu Lu; Siying Lv; Liqin Mo.

List of Supporting Information

Table S1. Comparison of various characteristics in the patient data (dead 3)

Table S2. Predictive performance of machine learning models (dead 1).

Table S3. Predictive performance of machine learning models (dead 2).

Table S4. Hyper-parametric optimization results.

Table S5. Range of reference values for each feature.

Table S6. SHAP Variable Importance Change Table

Table S1. Comparison of various characteristics in the patient data (dead 3)

	Data A (n=2610)		P value	Data B (n=1321)		P value
	Alive (n=2533)	Dead (n=77)		Alive (1284)	Dead (n=37)	
Age	63.76 ± 16.39	69.57 ± 14.19	<0.001	64.26 ± 16.03	65.60 ± 13.76	0.635
Gender			0.238			0.840
Male	1454	38		726	15	
Female	1079	38		558	10	
Temperature	36.89 (36.62, 37.25)	36.89 (36.59, 37.28)	0.985	37.15 (36.94, 37.41)	36.91 (36.67, 37.40)	0.017
Heart rate	88.30 ± 15.61	95.26 ± 17.56	<0.001	88.78 ± 15.68	91.07 ± 15.82	0.479
Systolic Blood Pressure	121.28 ± 17.98	108.30 ± 21.26	<0.001	122.31 ± 18.20	114.69 ± 14.00	0.013
Diastolic blood pressure	63.89 ± 13.13	64.46 ± 14.95	0.741	65.52 ± 11.66	63.38 ± 8.79	0.242
SO2	96.23 (94.71, 97.71)	94.57 (92.00, 97.30)	<0.001	96.28 ± 2.34	96.84 ± 2.47	0.270

	Data A (n=2610)		P value	Data B (n=1321)		P value
	Alive (n=2533)	Dead (n=77)		Alive (1284)	Dead (n=37)	
Breathing	20.82 (17.72, 24.23)	22.00 (18.19, 24.90)	0.294	20.98 ± 4.77	22.47 ± 7.66	0.343
Coronary disease	662	24	0.323	219	3	0.787
Diabetes	865	30	0.381	229	2	0.290
Hypertension	1787	58	0.364	226	0	0.014
Lung cancer	149	7	0.222	30	1	0.454
Activated partial thromboplastin time	32.50 (28.10, 42.30)	38.80 (31.00, 65.90)	<0.001	35.70 (27.85, 52.75)	32.90 (26.90, 40.00)	0.161
Alanine aminotransferase	28.00 (16.00, 59.00)	29.00 (19.00, 69.00)	0.210	33.00 (19.00, 65.00)	59.00 (18.00, 154.00)	0.082
Albumin	2.88 ± 0.60	2.75 ± 0.62	0.081	2.60 (2.10, 3.00)	2.27 (1.90, 2.70)	0.017
Aspartate aminotransfera	39.00 (23.00, 75.00)	42.00 (27.00, 94.00)	0.166	34.00 (20.00, 66.00)	55.00 (20.00, 166.00)	0.111

	Data A (n=2610)		P value	Data B (n=1321)		P value
	Alive (n=2533)	Dead (n=77)		Alive (1284)	Dead (n=37)	
se						
Blood sugar	126.00 (105.00, 158.67)	155.00 (117.00, 188.00)	<0.001	133.00 (110.00, 161.48)	132.00 (116.75, 175.38)	0.713
Calcium	8.30 (7.90, 8.75)	8.30 (7.80, 8.80)	0.856	8.20 (7.70, 8.70)	7.80 (7.15, 8.37)	0.060
Chlorine	103.29 ± 6.68	103.99 ± 7.46	0.414	105.06 ± 6.89	106.50 ± 5.00	0.168
Creatinine	1.00 (0.70, 1.80)	1.60 (1.10, 2.60)	<0.001	0.99 (0.65, 1.80)	1.16 (0.81, 1.98)	0.197
Hemoglobin	9.59 ± 1.76	8.99 ± 1.44	<0.001	9.40 (8.20, 11.10)	9.60 (8.30, 11.60)	0.263
International normalized	1.30 (1.10, 1.50)	1.60 (1.20, 2.17)	<0.001	1.30 (1.10, 1.60)	1.63 (1.30, 2.10)	0.003
Lactate dehydrogenase	295.00 (223.00, 426.00)	425.00 (285.00, 676.00)	<0.001	524.10 ± 396.26	310.40 ± 253.90	<0.001
Platelet	174.00 (103.33, 257.00)	148.00 (68.00, 209.00)	0.004	203.00 (141.00, 278.00)	157.00 (76.00, 198.00)	0.015
Potassium	3.90 (3.65, 4.28)	4.20 (3.77, 4.60)	<0.001	3.97 ± 0.57	4.00 ± 0.73	0.864

	Data A (n=2610)		P value	Data B (n=1321)		P value
	Alive (n=2533)	Dead (n=77)		Alive (1284)	Dead (n=37)	
Red blood cell	3.22 ± 0.64	3.02 ± 0.53	0.002	3.38 ± 0.66	3.34 ± 0.67	0.786
Sodium	139.68 ± 5.35	138.71 ± 6.15	0.177	140.00 (136.00, 143.00)	139.00 (132.00, 142.00)	0.206
Total bilirubin	0.70 (0.40, 1.50)	0.90 (0.30, 2.20)	0.573	0.57 (0.35, 0.90)	0.60 (0.40, 1.50)	0.461
White blood cell	10.37 (7.30, 14.30)	13.40 (8.50, 19.70)	<0.001	11.60 (8.40, 15.81)	16.50 (9.00, 18.40)	0.125
Respiratory type	2053	55	0.035	1216	25	0.637

Table S2. Predictive performance of machine learning models (dead 1)

Measure	LR	RF	LightGBM	XGBoost	MLP
Accuracy	0.943 (0.931-0.956)	0.963 (0.962-0.965)	0.962 (0.951-0.974)	0.964 (0.957-0.972)	0.966 (0.959-0.973)
Precision	0.227 (0.149-0.306)	0.367 (0.000-0.882)	0.443 (0.000-0.930)	0.551 (0.380-0.723)	0.548 (0.099-0.996)
Recall	0.467 (0.355-0.579)	0.091 (0.000-0.194)	0.310 (0.000-0.672)	0.500 (0.239-0.761)	0.348 (0.004-0.691)
F1 Score	0.302 (0.221-0.383)	0.139 (0.000-0.297)	0.353 (0.000-0.759)	0.509 (0.317-0.702)	0.413 (0.034-0.792)
AUC	0.829 (0.711-0.947)	0.882 (0.812-0.953)	0.872 (0.762-0.982)	0.867 (0.733-1.000)	0.796 (0.658-0.933)
Balanced ACC	0.719 (0.665-0.773)	0.544 (0.494-0.595)	0.652 (0.469-0.834)	0.746 （0.616-0.877）	0.672 (0.501-0.844)
MCC	0.307 (0.227-0.388)	0.173 (0.00-0.380)	0.356 (0.00-0.770)	0.510 (0.318-0.701)	0.424 (0.042-0.806)
Brier Score	0.035	0.017	0.018	0.014	0.014
Youden’s index	0.438	0.089	0.303	0.493	0.344

Table S3. Predictive performance of machine learning models (dead 2)

Measure	LSTM+LR	LSTM+RF	LSTM+LightGBM	LSTM+XGBoost	LSTM+MLP
Accuracy	0.958 (0.948-0.968)	0.966 (0.953-0.980)	0.967 (0.956-0.978)	0.968 (0.962-0.974)	0.968 (0.959-0.977)
Precision	0.593 (0.470-0.716)	0.693 (0.552-0.834)	0.702 (0.582-0.823)	0.720 (0.629-0.810)	0.728 (0.607-0.850)
Recall	0.646 (0.553-0.739)	0.646 (0.553-0.739)	0.625 (0.506-0.745)	0.625 (0.506-0.745)	0.615 (0.481-0.750)
F1 Score	0.613 (0.538-0.688)	0.666 (0.559-0.772)	0.659 (0.552-0.765)	0.664 (0.592-0.737)	0.661 (0.566-0.756)
AUC	0.847 (0.770-0.924)	0.858 (0.801-0.916)	0.863 (0.828-0.898)	0.867 (0.836-0.898)	0.827 (0.742-0.913)
Balanced ACC	0.807 (0.752-0.862)	0.821 (0.773-0.868)	0.809 (0.755-0.863)	0.810 (0.756-0.864)	0.801 (0.735-0.867)
MCC	0.608 (0.516-0.701)	0.676 (0.538-0.814)	0.637 (0.548-0.727)	0.645 (0.572-0.718)	0.646 (0.561-0.731)
Brier Score	0.039	0.031	0.033	0.032	0.032
Youden’s index	0.621	0.629	0.611	0.612	0.602

Table S4. Hyper-parametric optimization results (dead 3)

Model	Hyper-parameter space	Optimal hyper-parameter
LR	{'C': [0.001, 0.01, 0.1, 1, 10, 100], penalty': ['l1','l2'] 'solver': ['newton-cg', 'liblinear', 'sag', 'saga']}	{'C': 10, 'penalty': 'l2' 'solver': 'newton-cg'}
RF	{'n_estimators': range(100,300,10), 'max_depth': [None, 10, 20], 'min_samples_split': range(2,10,1) }	{'max_depth': 20, 'min_samples_split': 2, 'n_estimators': 280}
LightGBM	{'n_estimators': range(100,300,10), 'learning_rate': [0.1,0.01,1], 'num_leaves': [31, 50, 100], 'max_depth': range(1,10,1), 'min_child_samples': [5, 10, 20]}	{'learning_rate': 0.1, 'max_depth': 9, 'min_child_samples': 5, 'n_estimators': 290, 'num_leaves': 100}
XGBoost	{'learning_rate':[0.1,0.01,1], 'max_depth':range(1,10,1), 'min_child_weight':range(2,10,1), 'subsample':[0.5,0.7,1.0], 'colsample_bytree':[0.5,0.7,1.0], 'gamma':[0,1,5], 'n_estimators':range(30,300,5)}	{'colsample_bytree': 0.5, 'gamma': 1, 'learning_rate': 0.1, 'max_depth': 8, 'min_child_weight': 2, 'n_estimators': 290, 'subsample': 1.0}
MLP	{'activation':['relu','tanh'],	{'activation': 'tanh',

	'alpha':[0.0001,0.001,0.01,0.1],<br>'hidden_layer_sizes':[(50,),(100,),(200,)],<br>'learning_rate':['constant','adaptive']}	'alpha': 0.001,<br>'hidden_layer_sizes': (100,),<br>'learning_rate': 'constant'}

Table S5. Range of reference values for each feature

Variable	Lower limit	Upper limit	Variable	Lower limit	Upper limit
Activated partial thromboplastin time (sec)	25	36.5	Alanine aminotransferase (IU/L)	0	40
	22	35			
Albumin (g/dl)	3.5	5.2	Aspartate aminotransferase(IU/L)	0	40
Calcium (mg/dl)	8.4	10.3	Chlorine (mEq/L)	96	108
Creatinine (mg/dl)	0.4	1.1	Glucose (mg/dl)	70	100
Hemoglobin (g/dl)	13.7	17.5	International normalized ratio (%)	0.9	1.1
	12	16			
Lactate dehydrogenase (IU/L)	94	250	Oxygen saturation (%)		
Platelet (K/uL)	150	440	Potassium(mEq/L)	3.3	5.0
Prothrombin time (sec)	9.4	12.5	Red blood cell (K/uL)	4.6	6.1
				4.2	5.4
Sodium (mEq/L)	133	145	Total bilirubin (mg/dl)	0	1.5
White blood cell (K/uL)	4	11			

Table S6. SHAP Variable Importance Change Table

Outcome	Variable	Day 1	Day 2	Day 3	Day 4	Day 5	Day 6	Day 7	% Change	Direction	Peak Day
death_3d	spo2	0.001556	0.003790	0.003956					154.2%	**Increase**	Day 3
death_3d	ldh	0.001635	0.002228	0.004000					144.6%	**Increase**	Day 3
death_3d	sbp	0.001348	0.001958	0.004339					222.0%	**Increase**	Day 3
death_3d	dbp	0.001380	0.002196	0.002907					110.7%	**Increase**	Day 3
death_3d	potassium	0.001075	0.001314	0.003923					265.0%	**Increase**	Day 3
death_3d	glucose	0.002711	0.001477	0.001276					-52.9%	**Decrease**	Day 1
death_3d	heart_rate	0.001333	0.001465	0.002316					73.7%	**Increase**	Day 3
death_3d	temp	0.001658	0.001365	0.001901					14.6%	**Increase**	Day 3
death_3d	wbc	0.001235	0.001302	0.001734					40.5%	**Increase**	Day 3
death_3d	platelets	0.001708	0.001336	0.001136					-33.5%	**Decrease**	Day 1
death_5d	spo2	0.005392	0.003052	0.005873	0.006963	0.001532			-71.6%	**Decrease**	Day 4
death_5d	albumin	0.005713	0.003869	0.003140	0.002337	0.002071			-63.7%	**Decrease**	Day 1
death_5d	dbp	0.006758	0.003457	0.002439	0.001868	0.001850			-72.6%	**Decrease**	Day 1
death_5d	calcium	0.004841	0.003669	0.003241	0.002074	0.002046			-57.7%	**Decrease**	Day 1
death_5d	wbc	0.004078	0.003551	0.002856	0.002130	0.001927			-52.8%	**Decrease**	Day 1
death_5d	sodium	0.004408	0.002736	0.001910	0.002074	0.003325			-24.6%	**Decrease**	Day 1
death_5d	Age	0.004114	0.003229	0.002529	0.002143	0.001735			-57.8%	**Decrease**	Day 1
death_5d	heart_rate	0.004407	0.002423	0.002249	0.002379	0.002291			-48.0%	**Decrease**	Day 1

Outcome	Variable	Day 1	Day 2	Day 3	Day 4	Day 5	Day 6	Day 7	% Change	Direction	Peak Day
death_5d	sbp	0.002558	0.002073	0.002354	0.001690	0.004468			74.7%	**Increase**	Day 5
death_5d	Gender	0.003918	0.002833	0.001837	0.001664	0.002058			-47.5%	**Decrease**	Day 1
death_7d	spo2	0.001634	0.002166	0.002103	0.004415	0.001360	0.005568	0.005990	266.5%	**Increase**	Day 7
death_7d	dbp	0.002396	0.002498	0.002267	0.002005	0.001953	0.002209	0.002993	24.9%	**Increase**	Day 7
death_7d	albumin	0.002607	0.002152	0.001977	0.001587	0.001552	0.002150	0.003707	42.2%	**Increase**	Day 7
death_7d	heart_rate	0.002485	0.002424	0.002448	0.002280	0.001879	0.001695	0.002314	-6.9%	**Decrease**	Day 1
death_7d	platelets	0.002087	0.001894	0.002234	0.002184	0.002121	0.002483	0.002314	10.9%	**Increase**	Day 6
death_7d	hemoglobin	0.001723	0.002138	0.002053	0.002408	0.002303	0.002322	0.002331	35.3%	**Increase**	Day 4
death_7d	rbc	0.002445	0.002218	0.001741	0.001814	0.001902	0.002111	0.002848	16.5%	**Increase**	Day 7
death_7d	wbc	0.002147	0.002221	0.002303	0.001893	0.001708	0.001537	0.002270	5.7%	**Increase**	Day 3
death_7d	Age	0.002589	0.002479	0.002295	0.001922	0.001742	0.001380	0.001518	-41.4%	**Decrease**	Day 1
death_7d	ldh	0.002482	0.002231	0.002074	0.001721	0.001615	0.001520	0.002190	-11.7%	**Decrease**	Day 1

Note: higher values indicate greater contribution to model predictions.
